# Are microsporidia infections really increasing in HIV/AIDS patients or is it increased awareness?

**DOI:** 10.1093/inthealth/ihag019

**Published:** 2026-02-27

**Authors:** Sarman Singh

**Affiliations:** Advanced Centre for Chronic and Rare Diseases, New Delhi 110068, India

Editor,

I read with great interest the thought-provoking article by Asghari et al., published in the January 2026 issue of *International Health.*^1^ The authors present an updated systematic review and meta-analysis of microsporidia infections among people living with human immunodeficiency virus (HIV)/acquired immunodeficiency syndrome (AIDS), analysing studies published between 2017 and 2025. While the review addresses an important and neglected opportunistic infection, several conceptual and methodological issues merit further discussion.

The authors report screening 3318 studies, of which 39 studies encompassing 54 datasets were ultimately included. However, the rationale for restricting the analysis to publications from 2017 onwards is unclear and insufficiently justified. Microsporidia were recognised as clinically relevant pathogens only after the emergence of HIV/AIDS, particularly following the inclusion of AIDS-associated diarrhoea as an AIDS-defining condition in 1983. Reports of opportunistic parasitic infections, including microsporidia, began appearing in the mid-1980s. Excluding studies published prior to 2017 omits more than 3 decades of foundational epidemiological and clinical data.

A simple PubMed search using the authors’ keywords reveals that between 1985 and 2016, approximately 63 612 articles related to microsporidia were indexed, including at least 2531 original studies and clinical trials. This figure does not account for publications in non-indexed or regional journals, suggesting that the true body of literature is substantially larger. Importantly, a noticeable increase in publications occurred after 2010, likely reflecting improved awareness, better diagnostic tools and prolonged survival of people living with HIV due to effective antiretroviral therapy rather than a true surge in incidence. Excluding this extensive literature may therefore limit the interpretability and historical context of the findings.

I am also concerned by the authors’ assertion that ‘there is a possible increase in reported infections in recent years’, particularly after 2020. This conclusion does not appear to be supported by their own data. During the initial inclusion period (2017–2020), 18 studies were published, averaging 6 studies per year. In contrast, during the later period (2021–2025), 21 studies were published over 5 y, averaging only 4.2 studies per year. These figures do not indicate an increase in research output or reported infections.

More importantly, a key factor appears to have been overlooked: diagnostic methodology. Among studies published between 2017 and 2020, only 10 of 18 studies (55.5%) employed molecular methods for species identification. In contrast, during 2021–2025, 17 of 21 studies (80.9%) used molecular diagnostics—a substantial and statistically meaningful increase. This shift strongly suggests that improved diagnostic capacity and methodological rigor, rather than a true increase in infection rates, account for the increased detection and reporting of microsporidia. Consequently, attributing observed trends to an actual increase in infections may be misleading.

Another statement that warrants clarification is the authors’ expressed surprise that most studies originated from India and Iran. From a global research output perspective, this observation is not unexpected. While the USA historically led scientific publication output, this dominance has been challenged over the past 2 decades, particularly by China, which now leads globally in patents, publications and research output, especially after cuts in research funding by the Trump administration.^2,3^ India currently ranks 4th globally and Iran approximately 15th in terms of citable documents indexed in Scopus.^2,4^ In Asia, China remains the leading contributor, followed by India, which publishes more articles than Japan and South Korea, while Iran ranks fifth in the region. Iran has established itself as a significant producer of scientific literature, particularly in engineering, computer science and medical sciences, frequently ranking among the top 15–20 countries worldwide.^4^ Therefore, the predominance of studies from India and Iran likely reflects broader global research trends rather than an epidemiological anomaly.

Finally, I note that Table 3 stratifies patients by gender, CD4 count and age (<40 or ≥40 y) but does not distinguish between adult and paediatric populations. This omission is notable, as at least one included study (Kumar et al.^5^) focused exclusively on children. Given that clinical manifestations, immune responses and species distribution of microsporidia may differ substantially between paediatric and adult populations, a separate analysis or commentary on this subgroup would have added considerable value.

In conclusion, while the review by Asghari et al. draws attention to an important opportunistic infection, its conclusions regarding increasing infection rates should be interpreted cautiously. Greater emphasis on historical data, diagnostic evolution and population-specific analyses would strengthen future reviews and help distinguish true epidemiological trends from improved detection and reporting.

## Data Availability

Data are available in the article and references.

## Notes

**Comment on:** A global overview of microsporidia infection in HIV/AIDS patients: an updated systematic review and meta-analysis
